# Testimonials of Gratitude

**Published:** 1855-01

**Authors:** 


### TESTIMONIALS OF GRATITUDE.

The citizens of Savannah have recently held a meeting for the the purpose of expressing in a suitable and substantial manner, their gratitude to the Medical men of that city, and to those also who came from other cities to their relief during the recent epidemic visitation, for their faithful services, and their untiring efforts in behalf of the afflicted.

Drs. Redwood and Hamilton, of Mobile, and Dr. Cross, of New Orleans, were invited to attend, and before a large assemblage of citizens the Mayor presented them with a service of plate, each, for their noble services in the hour of their trial. These gentlemen above named had volunteered their services when the epidemic was sweeping away the inhabitants of the above city, and faithful to their high and holy purpose, they continued to labor through all the dark hours of its prevalence, in the face of ever threatening danger to themselves.

Resolutions were offered applauding the conduct of the resident physicians for the unfaltering perseverance in their duties, and another, which proposes the erection of a suitable monument to the memories of those who fell while contending with the disease.

We ask no better evidence of the liberality and intelligence of the citizens of Savannah than this noble demonstration of their gratitude, and every medical man of the entire country should cherish the recollection of that people, who, in this public manner, have so emphatically testified in behalf of medical science and the profession. *They* understand and appreciate the labors, sacrifices, and dangers, to which medical men are exposed; and show a liberal disposition to make it manifest to the world.

Had an enemy beseiged their town, and then entered by storm, scattering death and desolation in its onward march, those who heroically and manfully contended every step of its progress, and resisted every attempt at conquest, rapine and blood, would have had their names placed upon some enduring tablet to be perpetuated, honored, and revered in all coming time.

Noble as such an act would be, it is nevertheless no more than was performed by these self-sacrificing men, who, night and day, met the fearful enemy, nor knew what moment they themselves might be stricken down in death.

From the very depth of our hearts we pray that the citizens of Savannah may hereafter, during the existence of the lives of the present inhabitants at least, remain exempt from any further visitations of disease, that they may enjoy health and a long and happy life-time as they deserve.

